# Predictive value of a preoperative computed tomography-derived perifracture soft-tissue envelope index for fracture-related infection after open reduction and internal fixation of tibial plateau fractures

**DOI:** 10.3389/fmed.2026.1872629

**Published:** 2026-09-07

**Authors:** Jun Yang, Zhenhua Wu, Fang Shao, Leigang Qiao, Kun Li, Haicheng Yang

**Affiliations:** Department of Orthopedics, Ward I, Fengjie County People's Hospital, Fengjie County, Chongqing, China

**Keywords:** computed tomography, fracture-related infection, open reduction and internal fixation, Soft-tissue swelling, tibial plateau fracture

## Abstract

**Background:**

Fracture-related infection (FRI) is a major complication after open reduction and internal fixation (ORIF) of tibial plateau fractures. This study evaluated whether a preoperative computed tomography (CT)-derived soft-tissue envelope index (STEI) was associated with 12-month FRI and whether it provided incremental value for preoperative risk stratification.

**Methods:**

This retrospective single-center cohort study included 347 adults with unilateral closed tibial plateau fractures who underwent ORIF between January 2020 and December 2024. The STEI was defined as the mean ratio of soft-tissue area to osseous contour area at three axial levels located 5, 15, and 25 mm below the tibial plateau articular reference plane. FRI was adjudicated according to consensus confirmatory criteria. Every included patient had a dated, traceable, archived outcome record generated during clinical care after the 12-month anniversary of definitive ORIF. Non-FRI status required both the absence of any confirmatory criterion during the first postoperative year and explicit confirmation in a subsequent archived record. Logistic regression, receiver operating characteristic (ROC) curve analysis, calibration, decision curve analysis, and internal validation using 1,000 bootstrap resamples were performed.

**Results:**

The median follow-up duration was 12.8 months [interquartile range (IQR), 12.2–13.9], and 41 patients developed FRI (11.82%). The STEI was higher in the FRI group than in the non-FRI group (3.08 ± 0.52 vs. 2.51 ± 0.48; *P* < 0.001), and the interobserver intraclass correlation coefficient (ICC) was 0.946 [95% confidence interval (CI), 0.920–0.963]. The STEI remained independently associated with FRI [adjusted odds ratio (OR) per 0.10-unit increase, 1.21; 95% CI, 1.12–1.31; *P* < 0.001]. The combined preoperative model yielded an area under the ROC curve (AUC) of 0.865 (95% CI, 0.806–0.923), an optimism-corrected AUC of 0.847, and a calibration slope of 0.91, and provided greater net benefit across threshold probabilities of 5%−30%.

**Conclusion:**

The preoperative CT-derived STEI was independently associated with 12-month FRI after ORIF of closed tibial plateau fractures and added predictive information beyond clinical and fracture-related factors. Prospective multicenter external validation of both the index and the combined model is required.

## 1 Introduction

Tibial plateau fractures are common intra-articular fractures that may result from high-energy trauma or low-energy torsional injury, with underlying bone quality also influencing their occurrence. They are often accompanied by articular surface depression, metaphyseal comminution, and varying degrees of soft-tissue injury (1). Epidemiological data indicate that tibial plateau fractures account for a substantial proportion of fractures around the knee in adults, with marked heterogeneity in injury mechanisms and fracture morphology (2). Open reduction and internal fixation (ORIF) remains an important treatment for displaced tibial plateau fractures; its goals are to restore articular congruity and lower-limb alignment and to achieve stable fixation, thereby facilitating early functional rehabilitation (3). However, postoperative complications, particularly fracture-related infection (FRI), can substantially compromise fracture healing, fixation stability, and joint function (4).

Postoperative infection risk is jointly influenced by host status, local soft-tissue condition, fracture complexity, and surgical factors (5). The Schatzker, AO Foundation/Orthopedic Trauma Association (AO/OTA), and Oestern–Tscherne classification or grading systems help characterize the severity of osseous and soft-tissue injury; however, these categorical systems provide limited granularity, and interobserver agreement remains a concern in clinical practice (6). Different tibial plateau fracture classification systems also emphasize different aspects of fracture morphology and treatment planning (7). In patients with severe soft-tissue injury, staged fixation and careful assessment of local soft-tissue conditions may reduce complications (8). Soft-tissue coverage over the proximal tibia is limited, and marked swelling, tense fracture blisters, and impaired cutaneous microcirculation may increase the risk of wound complications and deep infection (9). Perioperative factors, including surgical timing, temporary external fixation, and fasciotomy, have also been associated with postoperative infection risk (10).

Preoperative computed tomography (CT) is routinely used to evaluate tibial plateau fractures. In addition to demonstrating articular depression, plateau widening, and column involvement, CT depicts the perifracture soft-tissue envelope. The soft-tissue envelope index (STEI) is calculated as the ratio of soft-tissue area to osseous contour area on the same axial image. Mathematically, this ratio may reduce the influence of body size, skeletal dimensions, and pixel scale on absolute area measurements; biologically, it may reflect circumferential expansion of the soft-tissue envelope caused by post-traumatic edema, hematoma, and inflammatory exudation. This study aimed to evaluate the association between a preoperative CT-derived STEI and 12-month FRI after ORIF of tibial plateau fractures and to develop a risk-prediction model integrating the STEI with preoperative clinical and fracture-related variables. Measurement reliability, model calibration, internal validation, and clinical net benefit were also evaluated.

## 2 Materials and methods

### 2.1 Study population

This retrospective single-center cohort study included adults with unilateral closed tibial plateau fractures who underwent ORIF at our hospital between January 2020 and December 2024. Reporting followed the Transparent Reporting of a multivariable prediction model for Individual Prognosis or Diagnosis + Artificial Intelligence (TRIPOD+AI) statement (11). The study was conducted in accordance with the Declaration of Helsinki and approved by the Ethics Committee of Fengjie County People's Hospital (approval no. AF/SS-02/02.0). The requirement for written informed consent was waived because only deidentified clinical and imaging data were used.

Eligibility criteria were: (1) age ≥18 years; (2) unilateral closed tibial plateau fracture confirmed by preoperative radiography and CT; (3) treatment with ORIF; (4) standardized preoperative thin-section CT performed at our hospital, with image quality adequate for STEI measurement; (5) complete data for the STEI and four prespecified non-STEI predictors (diabetes status, injury-to-surgery interval, albumin, and three-column involvement); and (6) at least one hospital outpatient record, routine structured telephone outcome-verification record, or clinical record from an outside institution after the 12-month anniversary of definitive ORIF that had been generated during clinical care, archived, dated, traceable, and sufficient to verify the FRI outcome during the first postoperative year. Exclusion criteria were: (1) open fracture; (2) pathological or old fracture; (3) previous ipsilateral knee infection, previous surgery of the ipsilateral proximal tibia, or marked deformity affecting measurement; (4) concomitant ipsilateral distal femoral, tibial shaft, distal tibial, or other injury that would interfere with local cross-sectional assessment; (5) severe immunodeficiency, long-term immunosuppressive therapy, or another definite active infection during the perioperative period; (6) missing data for the STEI or any of the four prespecified non-STEI predictors; and (7) absence of a verifiable outcome record after the 12-month postoperative anniversary.

The sample size was determined by the number of eligible patients who met the follow-up completeness criterion during the study period; no prospective sample-size calculation was performed. The final model contained five prespecified predictors. With 41 FRI events, the number of events per variable (EPV) was 8.2. Model stability was assessed by internal validation using 1,000 bootstrap resamples.

### 2.2 Perioperative management and fracture classification

After admission, all patients underwent a standardized trauma assessment, including evaluation of limb perfusion, neurologic function, local soft-tissue swelling, tense fracture blisters, and risk of acute compartment syndrome. Standard preoperative anteroposterior and lateral knee radiographs and CT of the tibial plateau were obtained, with multiplanar and three-dimensional reconstructions. Fractures were classified according to the Schatzker and AO/OTA 41 systems, and closed soft-tissue injuries were graded using the Oestern–Tscherne system. FRI diagnosis and outcome adjudication followed internationally recommended confirmatory criteria (12).

According to the three-column concept of the tibial plateau, posterior-column and three-column involvement were assessed on axial, coronal, and sagittal CT reconstructions. Posterior-column involvement was defined as extension of a fracture line into the posterior column, whereas three-column involvement required simultaneous involvement of the medial, lateral, and posterior columns. When local soft-tissue conditions precluded immediate definitive fixation, the limb was immobilized and elevated, with temporary external fixation when indicated, and definitive ORIF was delayed until soft-tissue recovery. Perioperative antimicrobial prophylaxis followed the institutional protocol and was generally administered intravenously 30–60 min before induction of anesthesia; repeat dosing was given for prolonged procedures or substantial blood loss. Microbiological and histopathological data were reviewed in accordance with FRI diagnostic recommendations (13).

### 2.3 Preoperative CT acquisition and STEI measurement

All patients underwent preoperative thin-section CT of the affected knee and proximal tibia. From January 2020 through December 2024, our institution used a prespecified trauma CT protocol for tibial plateau fractures, primarily with multidetector CT systems (SOMATOM Definition AS+, Siemens Healthineers; or Revolution EVO, GE HealthCare). Patients were positioned supine, with the knee extended as far as tolerated, the patella facing upward, and the foot in a neutral position; the scan range extended from the distal femur to the proximal tibia. Acquisition parameters included 120 kVp, automated tube-current modulation, a 512 × 512 matrix, and a 160–220-mm field of view. Axial images were reconstructed separately with a standard soft-tissue kernel and a high-resolution bone kernel, using a 1.0-mm section thickness and a 0.7–1.0-mm reconstruction interval. Soft-tissue area was traced at a window width of 400 Hounsfield units (HU) and a window level of 40 HU; osseous contours were traced at a window width of 1,500 HU and a window level of 450 HU. Areas were reported in mm^2^ by the planar measurement tool of the picture archiving and communication system (PACS). Acquisition and reconstruction parameters for all included scans fell within these prespecified ranges, and no protocol changes occurred during the study period. In cases with suspected infection but no clinical confirmatory sign, such as a sinus tract, fistula, or purulent drainage, imaging, laboratory, microbiological, and histopathological findings were reviewed within the current FRI diagnostic framework; however, the primary outcome remained based on confirmatory criteria (14).

The STEI is a study-specific CT-based quantitative metric constructed and named by our research team on the basis of established methods for CT cross-sectional tissue segmentation, cross-sectional area quantification, and lower-limb edema or volume measurement. Cadaveric validation studies have shown that CT can accurately quantify tissue cross-sectional area, and routine clinical CT can be used for standardized tissue segmentation and area measurement (15, 16). Post-traumatic edema, impaired lymphatic drainage, and changes in lower-limb volume can likewise be characterized using CT cross-sectional area or quantitative volumetric measurements (17–20). The study-specific components of the STEI were as follows: soft-tissue area was calculated by subtracting the combined tibial and fibular osseous contour area from the total cross-sectional area of the affected limb at the same level; soft-tissue area was normalized to osseous contour area to standardize for individual body size and local skeletal scale; and measurements at prespecified axial levels were combined with equal weighting. This normalization was intended to reduce the influence of body size and local skeletal scale on absolute soft-tissue area. All areas were exported in calibrated mm^2^ by the PACS tool.

Before measurements were performed in the main study cohort, an independent technical development set was assembled by stratified random sampling according to Schatzker classification from the prestudy imaging archive dated January 2018 through December 2019. The set comprised CT examinations from 60 adults with unilateral closed tibial plateau fractures: 20 each with Schatzker types I–II, III–IV, and V–VI. Candidate scans had to meet the same ranges for section thickness, reconstruction interval, and image quality as the main cohort; review of deidentified images was covered by the same ethics approval and waiver of written informed consent. This technical development set was independent of the 347-patient main cohort from 2020–2024 and was not part of either the 120-patient three-/five-level sensitivity subset or the 80-patient reliability subset. FRI outcomes and postoperative treatment information were not accessed during technical development. The initial candidate protocol comprised five axial levels—S5, S10, S15, S20, and S25—located 5, 10, 15, 20, and 25 mm below the tibial plateau articular reference plane. Six criteria for protocol finalization were prespecified before measurement: a case-level completion rate ≥95%; an interobserver intraclass correlation coefficient (ICC) ≥0.90; an agreement ICC between the three- and five-level protocols ≥0.95; an absolute paired mean difference < 0.05; an adjacent-level correlation coefficient *r* ≥0.85, indicating information redundancy; and a ≥25% reduction in measurement time with the three-level protocol relative to the five-level protocol. Two observers measured the candidate protocols while blinded to all outcome information. After the level-selection, boundary-tracing, and substitution rules had been finalized, all 60 development scans were rechecked using the final standard operating procedure (SOP).

Both the three- and five-level protocols were feasible in 60 of 60 cases under the prespecified rules [100.0%; 95% confidence interval (CI), 94.0%−100.0%]. Substitution with a level within ±1 mm was required for 4 of 180 candidate levels in the three-level protocol (2.2%; 95% CI, 0.6%−5.6%) and for 8 of 300 candidate levels in the five-level protocol (2.7%; 95% CI, 1.2%−5.2%; involving 7 patients). Pearson correlation coefficients between adjacent levels ranged from 0.86 to 0.93. Agreement between the three- and five-level protocols, evaluated using a two-way mixed-effects, absolute-agreement, single-measure ICC, was 0.986 (95% CI, 0.977–0.991). The paired difference (three-level minus five-level) was −0.009 ± 0.082 (95% CI, −0.030 to 0.012), with Bland–Altman 95% limits of agreement of −0.170 to 0.152. Interobserver ICCs for the three- and five-level protocols were 0.949 (95% CI, 0.916–0.969) and 0.954 (95% CI, 0.925–0.972), respectively. Observer 1 recorded the per-case measurement time from identification of the articular reference plane to output of the final STEI. Measurement times were 6.9 ± 1.2 min for the three-level protocol and 11.0 ± 1.6 min for the five-level protocol, with a paired difference of −4.1 ± 0.9 min (95% CI, −4.33 to −3.87). All prespecified criteria were met. Considering anatomic coverage, redundancy of information at adjacent levels, between-protocol agreement, and measurement efficiency, S5, S15, and S25 were selected as the equal-weighted three-level protocol for the primary analysis, whereas S10 and S20 were retained for the prespecified sensitivity analysis. The STEI name, formula, level locations, ±1-mm substitution rule, handling of detached small bone fragments, boundary-tracing rules, and primary protocol were locked before measurement of the main-cohort images and before linkage to FRI outcome data (Supplementary Table 1).

**Figure 1:**
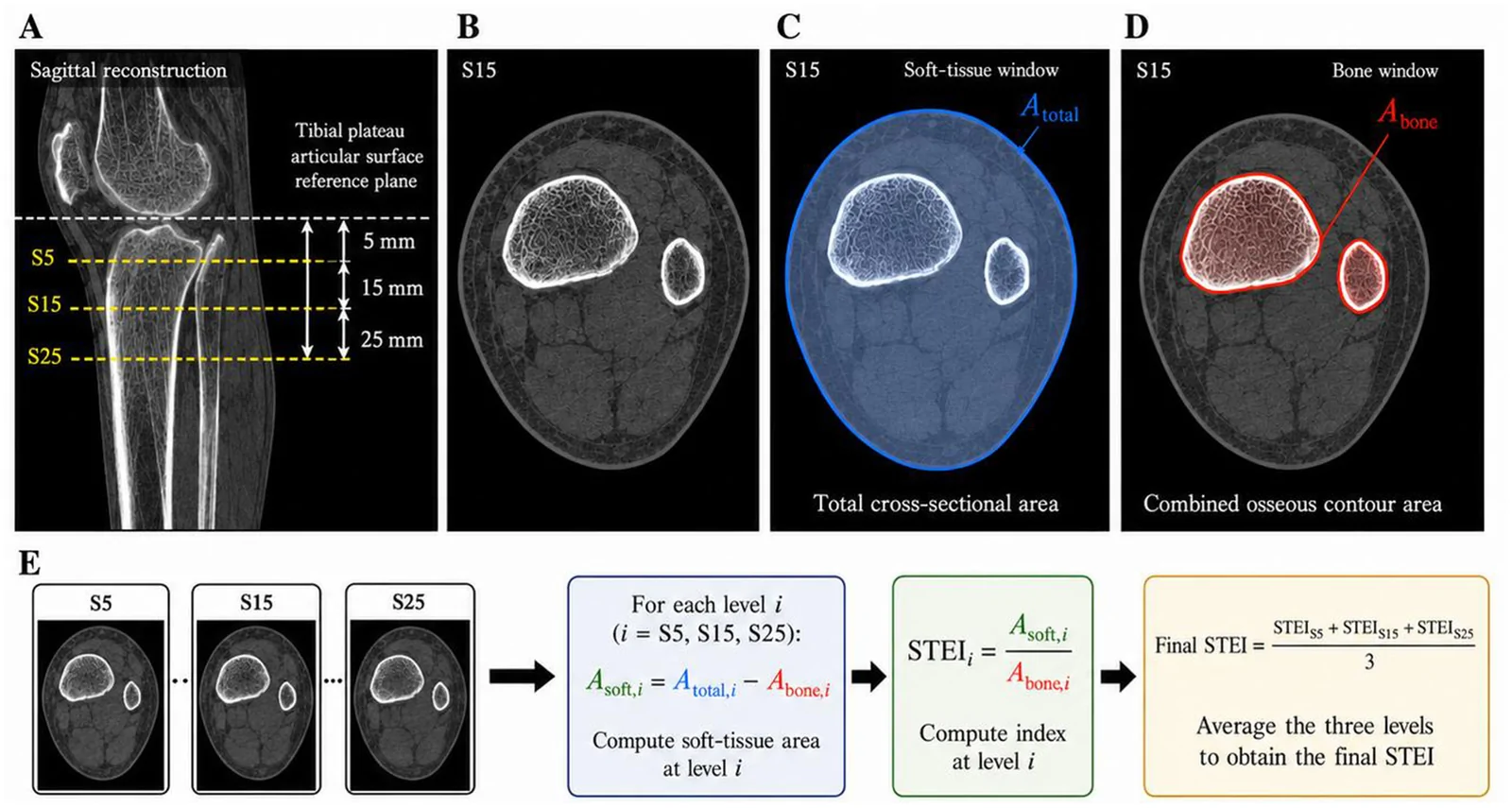
Standardized workflow for measuring the soft-tissue envelope index (STEI). **(A)** The tibial plateau articular reference plane is identified on a sagittal reconstruction, and three axial measurement levels are marked 5, 15, and 25 mm below this plane. **(B)** Axial computed tomography (CT) image at the S15 level. **(C)** The total cross-sectional area of the affected limb (*A*_total_) is traced along the outer skin contour. **(D)** The combined osseous contour area of the tibia and fibula (*A*_bone_) is traced along the outer cortical margins. **(E)** Soft-tissue area is calculated as *A*_soft_ = *A*_total_ – *A*_bone_; the index at each level is calculated as STEI_*i*_ = *A*_soft_/*A*_bone_; and the mean of the three levels is taken as the final STEI. *A*_total_, total cross-sectional area of the affected limb; *A*_bone_, combined tibial and fibular osseous contour area; *A*_soft_, soft-tissue area; S5, S15, and S25, axial levels 5, 15, and 25 mm below the tibial plateau articular reference plane, respectively.

The STEI at each level was calculated as follows: STEI_*i*_ = (*A*_total_ – *A*_bone_)/*A*_bone_ = *A*_soft_/*A*_bone_, where *A*_total_ is the total cross-sectional area of the affected limb, *A*_bone_ is the combined osseous contour area of the tibia and fibula on the same axial image, and *A*_soft_ = *A*_total_ – *A*_bone_. Measurements were performed as follows: (1) the tibial plateau articular reference plane was identified on coronal and sagittal reconstructions; when focal articular depression was present, an extrapolated line from the adjacent intact articular surface was used as the reference plane. (2) Three prespecified axial levels located 5, 15, and 25 mm below the reference plane were selected and designated S5, S15, and S25. When a target level contained a marked artifact or an incompletely visualized skin boundary, the closest level within ±1 mm that showed the entire skin contour was used. (3) On the soft-tissue window, *A*_total_ was traced continuously along the outer skin contour. (4) On the bone window, *A*_bone_ was traced along the outer cortical margins of the proximal tibia and fibula; detached small bone fragments were not separately included in the osseous contour area. (5) *A*_soft_ was calculated as *A*_total_ – *A*_bone_. (6) The index at each level was calculated as STEI_*i*_ = *A*_soft_/*A*_bone_. (7) The final STEI was the equal-weighted mean of the three level-specific measurements: STEI = (STEI_S5_ + STEI_S15_ + STEI_S25_)/3 (Figure 1). FRI microbiological findings were interpreted according to the current evidence framework (21).

In addition to the STEI, we recorded maximum articular depression depth, maximum plateau widening, posterior-column involvement, three-column involvement, degree of fracture comminution, and concomitant fibular head or proximal fibular fracture. CT morphology and treatment strategies for posterolateral and complex tibial plateau fractures were assessed with reference to recent literature (22). Preoperative imaging was used to characterize fracture morphology and soft-tissue status comprehensively (23). Fracture-morphology variables were categorized according to three-dimensional CT fracture mapping and morphometric frameworks (24).

The primary analysis used the first measurements completed and locked by Observer 1 for all 347 patients. Observer 1 was an attending orthopedic trauma surgeon with 8 years of experience in trauma imaging assessment; Observer 2 was a radiologist with 10 years of experience in musculoskeletal imaging. Observer 1 measured all 347 cases according to the locked SOP, whereas Observer 2 independently measured only the 80-patient reliability subset. After Observer 1 had completed and locked the initial measurements for the main cohort, an independent data manager who had not participated in CT measurement linked the FRI outcomes. A stratified random sample of 120 patients was then selected from the main cohort according to FRI outcome and Schatzker classification for the three-/five-level sensitivity analysis; 80 of these patients also constituted the reliability subset. Both observers received deidentified CT images without outcome labels. In the 80-patient reliability subset, both observers repeated the measurements 4 weeks later after the image order had been rerandomized. Interobserver reliability was assessed with a two-way random-effects, absolute-agreement, single-measure ICC; intraobserver reliability was assessed with a two-way mixed-effects, absolute-agreement, single-measure ICC. ICCs and 95% CIs were reported.

### 2.4 Clinical data collection

Patient data were obtained from the electronic medical record, surgical and anesthesia information system, nursing information system, laboratory information system, and follow-up records. When multiple preoperative measurements were available for the same variable, the value closest to definitive ORIF and with the most complete documentation was used. Collected variables included demographic characteristics (age, sex, body mass index [BMI], smoking history, and alcohol use), comorbidities, injury mechanism, injury-to-CT interval, injury-to-surgery interval, CT-to-surgery interval, tense fracture blisters, temporary external fixation, preoperative laboratory measures [white blood cell count, neutrophil percentage, C-reactive protein (CRP), hemoglobin, albumin, and venous blood glucose], surgical approach, fixation method, number of plates, operative time, bone grafting, estimated intraoperative blood loss, drain placement, fracture classification, and quantitative CT parameters.

The injury-to-CT interval was defined as the time from injury to the start of the first preoperative CT examination suitable for STEI measurement. The CT-to-surgery interval was defined as the time from that CT examination to definitive ORIF, and the injury-to-surgery interval was defined as the time from injury to definitive ORIF. For patients managed in stages, the date of definitive internal fixation was used in the statistical analysis. Patients with missing STEI data or missing data for any of the four prespecified non-STEI predictors (diabetes status, injury-to-surgery interval, albumin, and three-column involvement) were excluded by complete-case analysis. Reasons for exclusion were assigned to mutually exclusive categories according to a prespecified hierarchy; the amount and handling of missing data for each item are reported in the supplementary material (Supplementary Table 2).

### 2.5 Outcome definition and follow-up

The primary outcome was FRI occurring within 12 months after definitive ORIF. FRI was diagnosed when at least one of the following confirmatory criteria was met: (1) a sinus tract, fistula, or persistent wound breakdown communicating with the fracture site or implant; (2) definite purulent drainage identified during repeat debridement or revision surgery; (3) isolation of the same phenotypically indistinguishable microorganism from at least two independently collected deep-tissue or implant-related specimens; or (4) when histopathological data were available, acute inflammation or other histological evidence consistent with infection. A single positive culture from a deep specimen, elevated inflammatory markers, local erythema, swelling, warmth, pain, or imaging findings suspicious for infection were treated as suggestive criteria; the primary outcome was adjudicated according to confirmatory criteria.

Scheduled follow-up visits were at 2 weeks and 1, 3, 6, and 12 months after surgery. Actual follow-up duration was defined as the interval from definitive ORIF to the date of the last verifiable outcome record. For inclusion in the final analysis, a patient had to have at least one archived, dated, and traceable outcome-verification record generated during clinical care after the 12-month anniversary of definitive ORIF and sufficient to verify the FRI outcome during the first postoperative year. Verifiable sources comprised: (1) archived outpatient records from our hospital documenting wound status, a sinus tract or fistula, purulent drainage, infection-related reoperation, and imaging when indicated; (2) structured telephone outcome-verification records generated and archived through the hospital's routine follow-up process, together with complete review of the hospital outpatient, emergency, readmission, operative, microbiology, histopathology, and imaging systems; or (3) outside-institution outpatient or inpatient records, operative reports, and related test results obtained and archived during clinical care. This study involved only retrospective extraction and review of these pre-existing records. Standalone imaging or laboratory results were used only as corroborative evidence.

The hospital's routine structured telephone outcome-verification record used a dated standardized checklist addressing whether, during the first postoperative year, the patient had persistent wound breakdown or a sinus tract, purulent drainage, debridement or revision for suspected infection, deep-specimen culture or histopathological examination, infection-related readmission, or infection-related treatment at another institution. When a record contained suggestive information, the corresponding original medical records, operative reports, imaging, microbiological, or histopathological data were retrieved and adjudicated according to the FRI confirmatory criteria. Two orthopedic trauma surgeons experienced in FRI outcome adjudication and not involved in STEI measurement independently reviewed the primary outcome while blinded to STEI values and model outputs; disagreements were resolved by consensus after discussion.

FRI-positive status was defined as fulfillment of at least one FRI confirmatory criterion within 12 months after surgery. FRI-negative status required both of the following: (1) none of the available records during the first 12 postoperative months met an FRI confirmatory criterion; and (2) an archived, verifiable record after the 12-month anniversary explicitly confirmed that no infection-related event had occurred during the first postoperative year. Patients whose last verifiable record occurred within 12 months and for whom no later archived contact record or outside-institution data were available were excluded for incomplete follow-up.

### 2.6 Statistical analysis

Statistical analyses were performed using IBM SPSS Statistics version 26.0 (IBM Corp.) and R version 4.3.2 (R Foundation for Statistical Computing). Continuous-variable distributions were assessed using the Shapiro–Wilk test together with quantile–quantile (Q–Q) plots. Approximately normally distributed variables were presented as mean ± standard deviation and compared using Welch independent-samples *t* tests; nonnormally distributed variables were presented as median (interquartile range) and compared using the Mann–Whitney *U* test. Categorical variables were presented as number (percentage) and compared using the Pearson χ^2^ test; Fisher's exact test was used when expected cell counts were insufficient, and the Fisher–Freeman–Halton exact test was used for multicategory variables. Follow-up duration was presented as median (interquartile range) and range, and sources of outcome verification were presented as counts and percentages. Interobserver reliability was assessed with a two-way random-effects, absolute-agreement, single-measure ICC, whereas intraobserver reliability was assessed with a two-way mixed-effects, absolute-agreement, single-measure ICC. Bland–Altman analysis was also used to evaluate interobserver and between-protocol differences. Paired mean differences between the three- and five-level protocols were assessed with a paired *t* test. All tests were two-sided, and *P* < 0.05 was considered statistically significant.

Univariable logistic regression was performed with postoperative FRI as the dependent variable, and results are reported as odds ratios (ORs) with 95% confidence intervals (CIs). Predictor selection was based on a prespecified conceptual framework; no automated forward, backward, or stepwise procedure was used. Candidate variables were grouped into host factors, injury and soft-tissue status, laboratory measures, fracture-morphology variables, and indices of surgical complexity. A *P* value < 0.10 in univariable analysis was used only as a descriptive reference. The final model prioritized predictors that were available preoperatively, clinically relevant, representative of distinct pathophysiological domains, and minimally collinear. It contained five prespecified predictors; with 41 FRI events, the number of events per variable (EPV) was 8.2. Multicollinearity was assessed using the variance inflation factor (VIF), with VIF > 5 indicating substantial collinearity. Logit linearity for the STEI, injury-to-surgery interval, and albumin was assessed using restricted cubic splines with three knots placed at the 10th, 50th, and 90th percentiles; variables were entered as linear terms when no significant nonlinearity was detected.

Discrimination of the STEI and candidate models was assessed using receiver operating characteristic (ROC) curves, the area under the ROC curve (AUC), and 95% CIs. The Youden index was used to identify in-sample classification cutoffs, and correlated AUCs were compared with the DeLong test. To evaluate the incremental predictive value of the STEI, four prespecified nested models were compared: a clinical–fracture base model (diabetes, injury-to-surgery interval, albumin, and three-column involvement), the base model plus Oestern–Tscherne grade 2–3, the base model plus the STEI, and the base model plus both Oestern–Tscherne grade 2–3 and the STEI. Calibration was assessed using the Hosmer–Lemeshow goodness-of-fit test, calibration intercept, calibration slope, Brier score, and calibration plots.

Internal validation was performed with 1,000 bootstrap resamples. In each bootstrap sample, the model was refitted using the same five prespecified predictors, and performance was calculated in both the bootstrap sample and the original sample to estimate optimism in discrimination and calibration. Mean optimism was subtracted from the apparent performance of the original model to obtain optimism-corrected estimates. Decision curve analysis was used to evaluate clinical net benefit across threshold probabilities of 5%−30%. Pearson or Spearman correlation analysis was used to assess the associations of the STEI with BMI and the injury-to-CT interval. Sensitivity analyses added the injury-to-CT interval, or BMI and sex, to the final model.

## 3 Results

### 3.1 Patient selection

**Figure 2:**
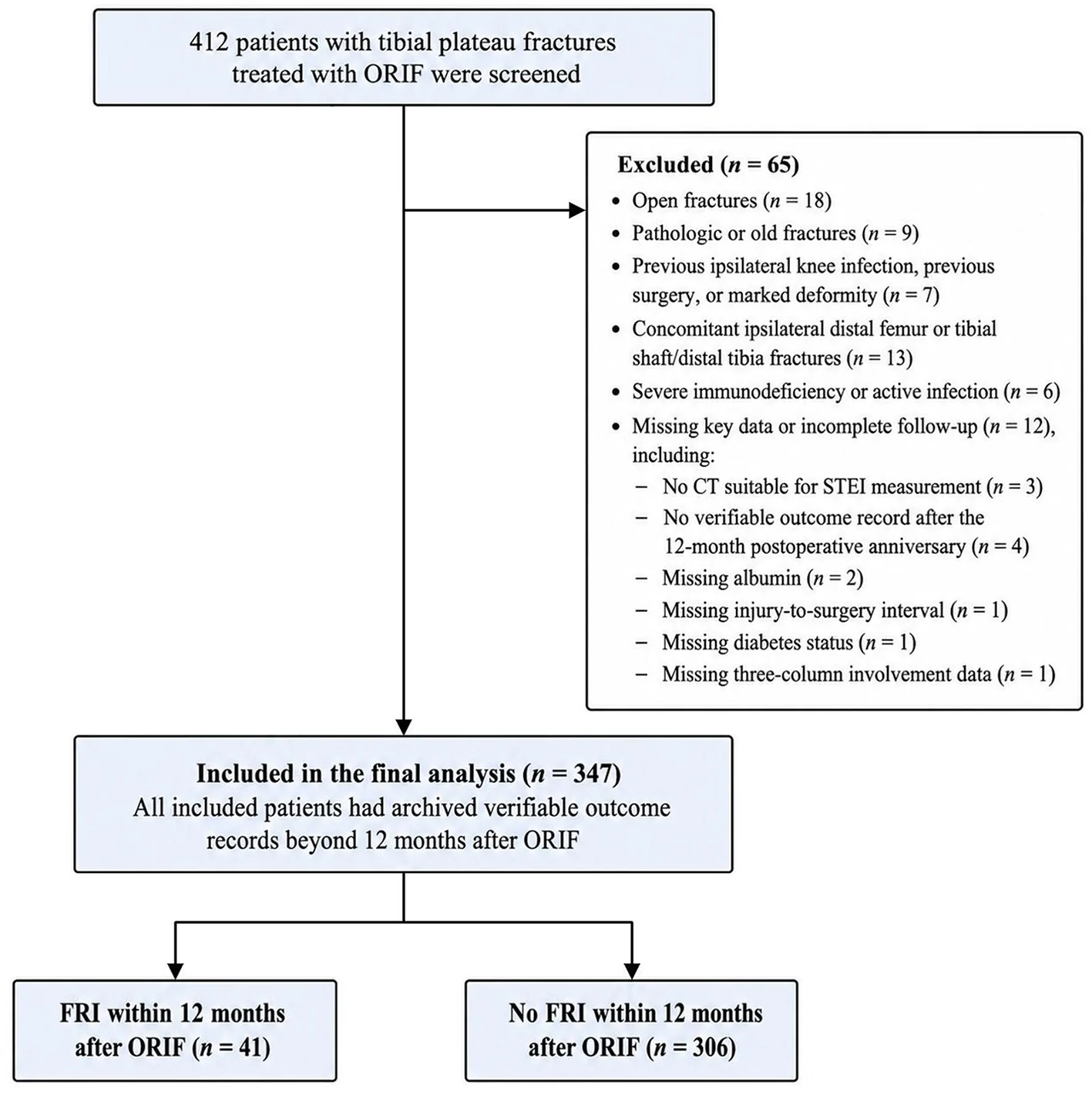
Patient-selection flowchart. During the study period, 412 patients with tibial plateau fractures treated with open reduction and internal fixation (ORIF) were screened. Exclusions comprised 18 open fractures, 9 pathological or old fractures, 7 cases with previous ipsilateral knee infection or surgery or marked deformity, 13 concomitant ipsilateral distal femoral or tibial shaft/distal tibial fractures, 6 cases with severe immunodeficiency or active infection during the perioperative period, and 12 cases with missing key data or incomplete follow-up. The latter group included 3 patients without computed tomography (CT) data suitable for soft-tissue envelope index (STEI) measurement, 4 without a verifiable outcome record after the 12-month postoperative anniversary, 2 with missing albumin, 1 with a missing injury-to-surgery interval, 1 with missing diabetes status, and 1 with missing three-column involvement data. The final analysis included 347 patients, all of whom had an archived, verifiable outcome record beyond 12 months after ORIF; 41 developed fracture-related infection (FRI) within 12 months and 306 did not. *n*, number of patients.

During the study period, 412 patients with tibial plateau fractures treated with ORIF were screened. Sixty-five were excluded: 18 with open fractures; 9 with pathological or old fractures; 7 with previous ipsilateral knee infection, surgery, or marked deformity; 13 with concomitant ipsilateral distal femoral or tibial shaft/distal tibial fractures; 6 with severe immunodeficiency or an active infection during the perioperative period; and 12 with missing key data or incomplete follow-up. The latter 12 comprised 3 patients without CT data suitable for STEI measurement, 4 without a verifiable outcome record after the 12-month postoperative anniversary, 2 with missing albumin, 1 with a missing injury-to-surgery interval, 1 with missing diabetes status, and 1 with missing three-column involvement data. The final cohort included 347 patients, all of whom had an archived, verifiable outcome record after the 12-month postoperative anniversary. Median follow-up was 12.8 months (interquartile range, 12.2–13.9; range, 12.1–18.7). Final verification was based on archived outpatient records from our hospital for 260 patients (74.93%), routine structured telephone outcome-verification records with complete review of the hospital information systems for 69 (19.88%), and traceable archived clinical records from outside institutions for 18 (5.19%). FRI occurred within 12 months in 41 patients (11.82%), whereas 306 did not develop FRI. For all 306 non-FRI patients, an archived record after 12 months explicitly confirmed the absence of an infection-related event during the first postoperative year. Four patients without such a post-12-month verification record were excluded for incomplete follow-up (Figure 2; Supplementary Table 3).

### 3.2 Baseline clinical characteristics, injury characteristics, and laboratory parameters

Compared with the non-FRI group, the FRI group had higher proportions of diabetes, high-energy injury, tense fracture blisters, and temporary external fixation; longer injury-to-CT, injury-to-surgery, and CT-to-surgery intervals; higher neutrophil percentages, CRP concentrations, and venous blood glucose levels; and lower albumin levels (all *P* < 0.05). Age, sex, BMI, smoking history, alcohol use, hypertension, coronary artery disease, chronic kidney disease, peripheral vascular disease, white blood cell count, and hemoglobin did not differ significantly between groups (all *P* > 0.05) (Table 1).

**Table 1:** Baseline clinical characteristics, injury characteristics, and laboratory parameters by FRI status.

Variable	FRI group (*n* = 41)	Non-FRI group (*n* = 306)	Statistic	*P* value
Age, y, mean ± SD	48.56 ± 13.71	46.88 ± 12.93	*t* = 0.74	0.462
Male sex, *n* (%)	31 (75.61)	208 (67.97)	*χ^2^* = 0.98	0.321
BMI, kg/m^2^, mean ± SD	26.83 ± 3.41	25.71 ± 3.18	*t* = 1.99	0.052
Smoking history, *n* (%)	22 (53.66)	132 (43.14)	*χ^2^* = 1.62	0.203
Alcohol use, *n* (%)	14 (34.15)	90 (29.41)	*χ^2^* = 0.39	0.534
Diabetes mellitus, *n* (%)	13 (31.71)	45 (14.71)	*χ^2^* = 7.51	0.006
Hypertension, *n* (%)	16 (39.02)	81 (26.47)	*χ^2^* = 2.83	0.093
Coronary artery disease, *n* (%)	5 (12.20)	18 (5.88)	Fisher's exact	0.170
Chronic kidney disease, *n* (%)	2 (4.88)	8 (2.61)	Fisher's exact	0.335
Peripheral vascular disease, *n* (%)	4 (9.76)	12 (3.92)	Fisher's exact	0.106
High-energy injury, *n* (%)	32 (78.05)	167 (54.58)	*χ^2^* = 8.14	0.004
Injury-to-CT interval, h, median (Q1, Q3)	13.0 (7.0, 25.0)	9.5 (5.0, 18.0)	*Z* = −2.11	0.035
Injury-to-surgery interval, d, median (Q1, Q3)	7.0 (5.0, 10.0)	5.0 (4.0, 7.0)	*Z* = −4.06	< 0.001
CT-to-surgery interval, d, median (Q1, Q3)	6.4 (4.4, 9.3)	4.6 (3.4, 6.4)	*Z* = −4.02	< 0.001
Tense fracture blisters, *n* (%)	18 (43.90)	51 (16.67)	*χ^2^* = 16.84	< 0.001
Temporary external fixation, *n* (%)	16 (39.02)	52 (16.99)	*χ^2^* = 11.14	0.001
White blood cell count, × 10^9^/L, mean ± SD	8.92 ± 2.61	8.17 ± 2.39	*t* = 1.74	0.087
Neutrophil percentage, %, mean ± SD	73.54 ± 9.06	69.81 ± 8.63	*t* = 2.49	0.016
C-reactive protein, mg/L, median (Q1, Q3)	21.4 (12.8, 36.9)	12.3 (7.1, 24.5)	*Z* = −3.13	0.002
Hemoglobin, g/L, mean ± SD	126.44 ± 15.79	129.82 ± 14.87	*t* = −1.30	0.201
Albumin, g/L, mean ± SD	37.55 ± 4.21	40.12 ± 3.84	*t* = −3.71	< 0.001
Venous blood glucose, mmol/L, median (Q1, Q3)	6.4 (5.7, 7.6)	5.8 (5.3, 6.6)	*Z* = −2.67	0.008

Approximately normally distributed continuous variables were compared using Welch independent-samples *t* tests; nonnormally distributed continuous variables were compared using the Mann–Whitney *U* test; categorical variables were compared using the χ^2^ test or Fisher's exact test. Multiple between-group comparisons were exploratory, and no multiplicity adjustment was applied. BMI, body mass index; CT, computed tomography; FRI, fracture-related infection; SD, standard deviation; Q1, first quartile; Q3, third quartile; *n*, number of patients; *P*, two-sided *P* value.

### 3.3 Fracture classification, CT parameters, and operative characteristics

Compared with the non-FRI group, the FRI group had higher proportions of Schatzker type V–VI fractures, AO/OTA type 41-C fractures, Oestern–Tscherne grade 2–3 injuries, posterior-column involvement, three-column involvement, severe comminution, and concomitant fibular head or proximal fibular fractures; the STEI, maximum articular depression depth, and maximum plateau widening were also greater. The FRI group more often underwent a combined approach, dual-plate fixation, bone grafting or bone-substitute augmentation, and drain placement, and had longer operative times and greater estimated intraoperative blood loss (all *P* < 0.05) (Table 2).

**Table 2:** Fracture classification, CT parameters, and operative characteristics by FRI status.

Variable	FRI group (*n* = 41)	Non-FRI group (*n* = 306)	Statistic	*P* value
Schatzker classification			*χ^2^* = 12.29	0.002
Type I–II, *n* (%)	4 (9.76)	67 (21.90)		
Type III–IV, *n* (%)	9 (21.95)	118 (38.56)		
Type V–VI, *n* (%)	28 (68.29)	121 (39.54)		
AO/OTA classification			*χ^2^* = 13.47	< 0.001
Type 41-B, *n* (%)	10 (24.39)	168 (54.90)		
Type 41-C, *n* (%)	31 (75.61)	138 (45.10)		
Oestern–Tscherne grade			*χ^2^* = 17.92	< 0.001
Grade 0–1, *n* (%)	15 (36.59)	214 (69.93)		
Grade 2–3, *n* (%)	26 (63.41)	92 (30.07)		
STEI, mean ± SD	3.08 ± 0.52	2.51 ± 0.48	*t* = 6.65	< 0.001
Maximum articular depression depth, mm, mean ± SD	8.62 ± 2.86	6.47 ± 2.55	*t* = 4.58	< 0.001
Maximum plateau widening, mm, mean ± SD	7.86 ± 2.49	5.81 ± 2.17	*t* = 5.02	< 0.001
Posterior-column involvement, *n* (%)	28 (68.29)	125 (40.85)	*χ^2^* = 11.05	0.001
Three-column involvement, *n* (%)	20 (48.78)	74 (24.18)	*χ^2^* = 11.08	0.001
Fracture comminution			*χ^2^* = 15.23	< 0.001
Mild, *n* (%)	6 (14.63)	91 (29.74)		
Moderate, *n* (%)	11 (26.83)	128 (41.83)		
Severe, *n* (%)	24 (58.54)	87 (28.43)		
Concomitant fibular head or proximal fibular fracture, *n* (%)	18 (43.90)	84 (27.45)	*χ^2^* = 4.71	0.030
Surgical approach			Fisher–Freeman–Halton	0.013
Anterolateral approach, *n* (%)	18 (43.90)	199 (65.03)		
Posteromedial approach, *n* (%)	3 (7.32)	26 (8.50)		
Combined approach, *n* (%)	20 (48.78)	81 (26.47)		
Fixation method			Fisher–Freeman–Halton	0.022
Anatomic locking plate, *n* (%)	17 (41.46)	193 (63.07)		
Buttress plate, *n* (%)	3 (7.32)	19 (6.21)		
Dual-plate fixation, *n* (%)	21 (51.22)	94 (30.72)		
Bone grafting or bone-substitute augmentation, *n* (%)	20 (48.78)	83 (27.12)	*χ^2^* = 8.12	0.004
Operative time, min, mean ± SD	142.3 ± 36.1	121.4 ± 30.8	*t* = 3.54	0.001
Estimated intraoperative blood loss, mL, median (Q1, Q3)	180 (120, 260)	130 (90, 200)	*Z* = −3.59	< 0.001
Drain placement, *n* (%)	28 (68.29)	151 (49.35)	*χ^2^* = 5.20	0.023

Approximately normally distributed continuous variables were compared using Welch independent-samples *t* tests; nonnormally distributed continuous variables were compared using the Mann–Whitney *U* test. Surgical approach and fixation method were compared using the Fisher–Freeman–Halton exact test. AO/OTA, AO Foundation/Orthopedic Trauma Association; CT, computed tomography; FRI, fracture-related infection; STEI, soft-tissue envelope index; SD, standard deviation; Q1, first quartile; Q3, third quartile; *n*, number of patients; *P*, two-sided *P* value.

### 3.4 FRI confirmatory criteria and STEI measurement reliability

All 41 FRI cases met at least one confirmatory criterion: 8 had a sinus tract, fistula, or persistent wound breakdown; 17 had definite purulent drainage identified at reoperation; 31 yielded the same phenotypically indistinguishable microorganism from at least two independent deep-tissue or implant-related specimens; and 11 had histopathological evidence of acute inflammation. Criteria could overlap (Supplementary Table 4).

In the 80-patient reliability subset, the interobserver ICC for the STEI was 0.946 (95% CI, 0.920–0.963), and the intraobserver ICCs for Observers 1 and 2 were 0.972 (95% CI, 0.958–0.982) and 0.958 (95% CI, 0.936–0.973), respectively. The mean interobserver difference was 0.01, the standard deviation of the differences was 0.169, and the Bland–Altman 95% limits of agreement were −0.32 to 0.34 (Supplementary Table 5). The STEI showed weak positive correlations with BMI and the injury-to-CT interval. Its effect estimate remained stable after addition of the injury-to-CT interval or after addition of BMI and sex. Restricted cubic splines showed no significant nonlinearity for the STEI, injury-to-surgery interval, or albumin (Supplementary Table 6). In the 120-patient sensitivity subset, the three- and five-level STEI protocols showed high agreement, and their AUCs did not differ significantly (Supplementary Table 7).

### 3.5 Univariable and multivariable logistic regression analyses of postoperative FRI

Univariable logistic regression showed that diabetes, high-energy injury, a longer injury-to-surgery interval, tense fracture blisters, temporary external fixation, a higher neutrophil percentage, higher CRP, Schatzker type V–VI, AO/OTA type 41-C, Oestern–Tscherne grade 2–3, lower albumin, higher venous blood glucose, a higher STEI, greater maximum articular depression depth, greater maximum plateau widening, posterior-column involvement, three-column involvement, severe comminution, a concomitant fibular head or proximal fibular fracture, a combined approach, bone grafting or bone-substitute augmentation, dual-plate fixation, a longer operative time, greater estimated intraoperative blood loss, and drain placement were associated with FRI (all *P* < 0.05) (Table 3).

**Table 3:** Univariable logistic regression analysis of postoperative FRI.

Variable	Wald *χ^2^*	OR	95% CI	*P* value
Diabetes mellitus	7.08	2.69	1.30–5.59	0.008
High-energy injury	7.57	2.96	1.37–6.41	0.006
Injury-to-surgery interval (per 1-day increase)	12.46	1.14	1.06–1.23	< 0.001
Tense fracture blisters	15.19	3.91	1.97–7.77	< 0.001
Temporary external fixation	10.34	3.13	1.56–6.26	0.001
Neutrophil percentage (per 1% increase)	6.04	1.05	1.01–1.09	0.014
C-reactive protein (per 10-mg/L increase)	12.92	1.17	1.07–1.27	< 0.001
Schatzker type V–VI (vs. type I–IV)	11.25	3.29	1.64–6.61	0.001
AO/OTA type 41-C (vs. type 41-B)	12.13	3.77	1.79–7.97	< 0.001
Oestern–Tscherne grade 2–3 (vs. grade 0–1)	16.11	4.03	2.04–7.97	< 0.001
Albumin (per 1-g/L increase)	15.07	0.85	0.78–0.92	< 0.001
Venous blood glucose (per 1-mmol/L increase)	7.76	1.29	1.08–1.54	0.005
STEI (per 0.10-unit increase)	35.64	1.27	1.17–1.37	< 0.001
Maximum articular depression depth (per 1-mm increase)	14.31	1.24	1.11–1.39	< 0.001
Maximum plateau widening (per 1-mm increase)	16.49	1.29	1.14–1.46	< 0.001
Posterior-column involvement	10.25	3.12	1.55–6.26	0.001
Three-column involvement	10.37	2.99	1.53–5.81	0.001
Severe comminution (vs. mild/moderate)	13.80	3.55	1.82–6.94	< 0.001
Concomitant fibular head or proximal fibular fracture	4.57	2.07	1.06–4.03	0.032
Combined approach (vs. noncombined approach)	8.27	2.65	1.36–5.13	0.004
Bone grafting or bone-substitute augmentation	7.73	2.56	1.32–4.96	0.005
Dual-plate fixation (vs. non-dual-plate fixation)	6.58	2.37	1.23–4.58	0.010
Operative time (per 10-min increase)	10.84	1.13	1.05–1.22	0.001
Estimated intraoperative blood loss (per 50-ml increase)	8.88	1.18	1.06–1.32	0.003
Drain placement	5.01	2.21	1.10–4.43	0.025

ORs and 95% CIs for binary variables were calculated from the original 2 × 2 frequencies; intervals for continuous variables were derived from the corresponding logistic regression coefficient and standard error. The STEI was analyzed per 0.10-unit increase. AO/OTA, AO Foundation/Orthopedic Trauma Association; FRI, fracture-related infection; STEI, soft-tissue envelope index; OR, odds ratio; CI, confidence interval; *P*, two-sided *P* value.

ORs and 95% CIs for binary variables were calculated from the original 2 × 2 frequencies; intervals for continuous variables were derived from the corresponding logistic regression coefficient and standard error. The STEI was analyzed per 0.10-unit increase. AO/OTA, AO Foundation/Orthopedic Trauma Association; FRI, fracture-related infection; STEI, soft-tissue envelope index; OR, odds ratio; CI, confidence interval; *P*, two-sided *P* value.

The final multivariable model was determined according to the prespecified conceptual framework, preoperative availability, collinearity diagnostics, and the number of events. It included diabetes, injury-to-surgery interval, albumin, the STEI, and three-column involvement. A higher STEI, diabetes, a longer injury-to-surgery interval, and three-column involvement were associated with higher FRI risk, whereas higher albumin was associated with lower FRI risk. VIFs ranged from 1.18 to 2.34 (Table 4).

**Table 4:** Multivariable logistic regression analysis of postoperative FRI and prediction-model coefficients.

Variable	β	SE	Wald *χ^2^*	OR	95% CI	*P* value	VIF
Diabetes mellitus	0.859	0.380	5.11	2.36	1.12–4.97	0.024	1.22
Injury-to-surgery interval (per 1-day increase)	0.086	0.035	6.04	1.09	1.02–1.17	0.014	1.39
Albumin (per 1-g/L increase)	−0.105	0.042	6.25	0.90	0.83–0.98	0.012	1.18
STEI (per 0.10-unit increase)	0.1906	0.0402	22.48	1.21	1.12–1.31	< 0.001	2.34
Three-column involvement	0.880	0.401	4.82	2.41	1.10–5.29	0.028	1.86

The β coefficient, SE, and OR for the STEI are reported per 0.10-unit increase; in the prediction equation, the STEI is entered per 1.0-unit increase, corresponding to a coefficient of 1.906. FRI, fracture-related infection; STEI, soft-tissue envelope index; β, logistic regression coefficient; SE, standard error; OR, odds ratio; CI, confidence interval; VIF, variance inflation factor; *P*, two-sided *P* value.

### 3.6 Predictive performance of the STEI and combined models

The STEI-only model had an AUC of 0.789 (95% CI, 0.716–0.862), and the in-sample classification cutoff determined by the Youden index was 2.85. The clinical–fracture base model had an AUC of 0.802. After addition of the Oestern–Tscherne grade 2–3 indicator, the AUC was 0.824, which did not differ significantly from that of the base model. The final combined model incorporating the STEI had an AUC of 0.865 (95% CI, 0.806–0.923), which was higher than those of both the base model and the base model plus the Oestern–Tscherne grade 2–3 indicator. The model incorporating both the Oestern–Tscherne grade 2–3 indicator and the STEI had an AUC of 0.869, which did not differ significantly from that of the final combined model (Table 5).

**Table 5:** Predictive performance and incremental value of the STEI across models.

Model	AUC (95% CI)	In-sample cutoff	Sensitivity (%)	Specificity (%)	Youden index	Brier score	Comparison	*P* value
STEI-only model	0.789 (0.716–0.862)	2.85	78.05	70.59	0.486	0.089	Reference	—
Clinical–fracture base model	0.802 (0.729–0.875)	0.13	75.61	75.16	0.508	0.084	Reference	—
Base model + Oestern–Tscherne grade 2–3	0.824 (0.754–0.894)	0.13	78.05	76.47	0.545	0.081	Vs. base model	0.214
Base model + STEI (final combined model)	0.865 (0.806–0.923)	0.14	80.49	79.08	0.596	0.072	vs. base model; vs. base model + Oestern–Tscherne grade 2–3	0.006; 0.031
Base model + Oestern–Tscherne grade 2–3 + STEI	0.869 (0.811–0.927)	0.14	82.93	77.78	0.607	0.071	Vs. final combined model	0.610

Cutoffs were determined by the Youden index in the current sample and describe in-sample classification performance. Correlated ROC curves were compared using the DeLong test. AUC, area under the receiver operating characteristic curve; CI, confidence interval; ROC, receiver operating characteristic; STEI, soft-tissue envelope index; *P*, two-sided *P* value.

**Figure 3:**
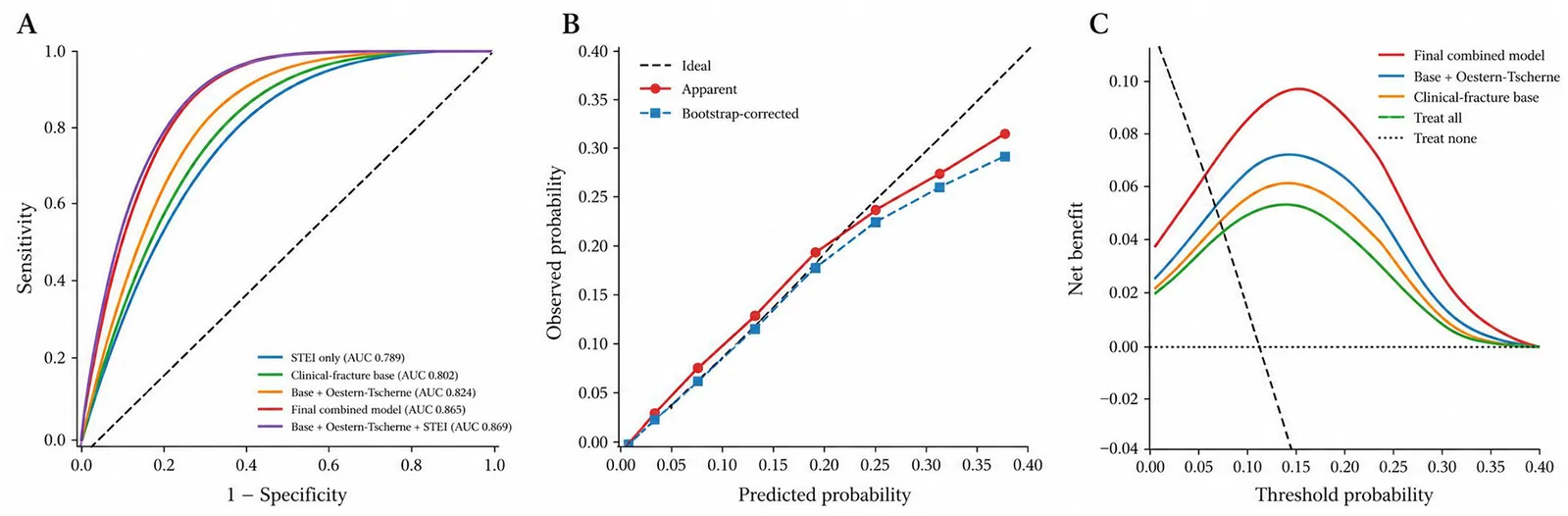
Model discrimination, calibration, and clinical net benefit. **(A)** Receiver operating characteristic (ROC) curves for the soft-tissue envelope index (STEI)-only model, clinical–fracture base model, base model plus the Oestern–Tscherne grade 2–3 indicator, final combined model, and base model plus the Oestern–Tscherne grade 2–3 indicator plus STEI. **(B)** Calibration plot for the final combined model; the dashed line indicates ideal calibration, the solid line indicates apparent calibration, and the dash-dotted line indicates bootstrap-corrected calibration. **(C)** Decision curve analysis (DCA) shows that the final combined model provides greater net benefit across threshold probabilities of 5%−30% and outperforms the treat-all and treat-none strategies. AUC, area under the ROC curve; Oestern–Tscherne grade, closed soft-tissue injury grade.

The predicted probability from the final combined model was calculated as follows: logit(*P*) = −3.610 + 0.859 × diabetes + 0.086 × injury-to-surgery interval (d) – 0.105 × albumin (g/L) + 1.906 × STEI + 0.880 × three-column involvement. Diabetes and three-column involvement were coded as yes = 1 and no = 0, and *P* = 1/[1 + exp(–logit(*P*))]. The Hosmer–Lemeshow goodness-of-fit test yielded *P* = 0.627. After internal validation with 1,000 bootstrap resamples, the optimism-corrected AUC was 0.847, the calibration intercept was −0.02, the calibration slope was 0.91, and the Brier score was 0.076 (Table 6; Figure 3). Decision curve analysis showed greater net benefit for the final combined model across threshold probabilities of 5%−30% (Figure 3).

**Table 6:** Calibration and internal validation metrics reported in accordance with the TRIPOD+AI statement.

Metric	AUC	Calibration intercept	Calibration slope	Brier score	Description
Apparent model	0.865	0.00	1.00	0.072	Hosmer–Lemeshow *χ^2^* = 6.18; *P* = 0.627
Bootstrap-corrected model (1,000 resamples)	0.847	−0.02	0.91	0.076	Refitted with the same five prespecified predictors in each resample and corrected for optimism
Null model	0.500	—	—	0.104	Calculated from the 11.82% event rate

AUC, area under the receiver operating characteristic curve; TRIPOD+AI, Transparent Reporting of a multivariable prediction model for Individual Prognosis Or Diagnosis + Artificial Intelligence; Hosmer–Lemeshow χ^2^, Hosmer–Lemeshow goodness-of-fit statistic; *P*, two-sided *P* value. Ideal values are 0 for the calibration intercept and 1 for the calibration slope.

## 4 Discussion

This study included 347 patients with closed tibial plateau fractures treated with ORIF, among whom the 12-month FRI incidence was 11.82%. This incidence lies within the range recently reported after surgical treatment of closed tibial plateau fractures (25). Orthopedic surgical-site and deep infections are jointly associated with host metabolic status, injury severity, local soft-tissue condition, surgical complexity, and microbiological factors (26, 27). Previous prediction studies of deep infection after tibial plateau and pilon fractures have likewise shown that preoperatively available variables can support infection-risk stratification (28).

The STEI quantifies the local soft-tissue envelope as a continuous variable. It was higher in the FRI group than in the non-FRI group, and each 0.10-unit increase was associated with higher FRI risk. Adding the STEI to the clinical–fracture base model increased the AUC from 0.802 to 0.865, and its incremental contribution exceeded that of adding the Oestern–Tscherne grade alone. Previous orthopedic infection-prediction studies have shown that combining clinical, imaging, and fracture-complexity information can improve individual risk estimates (29, 30). Because the pathogenesis and treatment pathways of FRI are multifactorial, preoperative risk assessment should consider both local soft-tissue status and systemic host factors (31).

Diabetes was independently associated with FRI, consistent with existing evidence linking perioperative dysglycemia to adverse orthopedic outcomes and with plausible immune and microcirculatory mechanisms (32). Higher albumin was associated with lower FRI risk; previous orthopedic trauma studies have linked hypoalbuminemia to adverse outcomes, indirectly supporting interpretation in terms of nutritional and systemic status (33). The association with a longer injury-to-surgery interval may reflect a combination of greater soft-tissue injury severity, the need for staged treatment, and a longer duration of inflammation; its causal direction requires prospective confirmation. Three-column involvement was independently associated with FRI, suggesting that complex fracture morphology may increase infection risk through higher-energy injury, more extensive soft-tissue dissection, longer operative time, and more complex fixation requirements.

We report the complete logistic regression equation, regression coefficients, intercept, calibration metrics, and internal-validation results. Evaluation of prediction models should consider discrimination, calibration, control of overfitting, and reproducibility (34). The bootstrap-corrected AUC of 0.847 and calibration slope of 0.91 indicate some optimism in the current sample; generalizability therefore requires independent external validation (35). Decision curve analysis demonstrated greater net benefit for the final combined model across threshold probabilities of 5%−30%. Such analysis complements AUC and calibration by evaluating the clinical value of decisions across different thresholds (36, 37).

The primary outcome was adjudicated using FRI confirmatory criteria, which may improve the specificity of deep-FRI classification. FRI treatment and prognosis are jointly influenced by the timing of infection, microbiology, fracture stability, soft-tissue coverage, and host status; the 12-month postoperative outcome window was selected to capture clinically meaningful infections requiring further treatment (38). The current FRI framework emphasizes a sinus tract or fistula, purulent drainage, repeatedly positive deep-specimen cultures, and histopathological evidence (39). The timing of surgical intervention after FRI diagnosis should be determined according to infection duration, fixation stability, soft-tissue condition, and host status (40). FRI occurrence and management reflect interactions among fixation stability, microbiology, soft-tissue coverage, and host status (41), and soft-tissue management requires adequate debridement, reliable coverage, and multidisciplinary collaboration (42).

This study has several limitations. First, the retrospective single-center design limits external generalizability, and model transportability requires validation in independent cohorts. Second, the STEI depends on manual contouring and therefore remains susceptible to operator-related error. Third, scanner type, reconstruction kernel, section thickness and interval, window settings, degree of knee extension, and limb rotation may affect measurement, and acquisition differences across institutions may reduce between-center reproducibility. Fourth, post-traumatic soft-tissue swelling changes over time, so the injury-to-CT interval may cause residual confounding. Fifth, routine preoperative CT did not systematically include the corresponding contralateral levels, precluding calculation of an injured-to-uninjured limb ratio. Sixth, complete-case analysis may introduce selection bias. Although every included patient had an archived, verifiable outcome record beyond 12 months after surgery, routine structured telephone records may be affected by recall bias, the completeness of archived outside-institution records may vary, and residual outcome misclassification cannot be excluded. Seventh, the final model was based on only 41 FRI events, with an EPV of 8.2 for five predictors, so regression coefficients and performance estimates may remain unstable. Eighth, the multiple between-group comparisons of baseline and operative variables were exploratory and were not adjusted for multiplicity. Prospective multicenter studies should use standardized CT protocols, prespecified scan-time windows, automated or semiautomated segmentation, model updating, and clinical-impact assessment.

## 5 Conclusions

In this single-center cohort, the preoperative CT-derived STEI was independently associated with 12-month FRI after ORIF of closed tibial plateau fractures and showed high interobserver and intraobserver reliability in the reliability subset. A combined model integrating the STEI, diabetes, injury-to-surgery interval, albumin, and three-column involvement demonstrated good discrimination and acceptable calibration on internal validation and may provide supplementary information for preoperative infection-risk stratification. The interinstitutional reproducibility of the STEI, the in-sample classification cutoff, and the clinical generalizability of the combined model require prospective multicenter external validation under standardized CT protocols.

## Data Availability

The original contributions presented in the study are included in the article/Supplementary material, further inquiries can be directed to the corresponding author.
