# Supplementary material for: Predictive value of a preoperative computed tomography-derived perifracture soft-tissue envelope index for fracture-related infection after open reduction and internal fixation of tibial plateau fractures
Source: Front Med (Lausanne). 2026 Sep 7;13:1872629. doi: 10.3389/fmed.2026.1872629 (PMC13590323; doi:10.3389/fmed.2026.1872629)
Supplement: Supplementary file 1 [file Data_Sheet_1.docx]

**Supplementary Material**

**Supplementary Table 1.** Pre-application technical evaluation of the STEI in the independent technical development set

| **Metric** | **Three-level protocol** | **Five-level protocol/other result** | **95% CI/range** | **Description** |
| --- | --- | --- | --- | --- |
| Technical development set | 60 patients | 20 each with Schatzker types I–II, III–IV, and V–VI | January 2018 to December 2019 | Covered by the same ethics approval and waiver of informed consent; independent of the 347-patient main cohort, 120-patient sensitivity subset, and 80-patient reliability subset |
| Measurement feasible | 60/60 (100.0%) | 60/60 (100.0%) | 94.0%–100.0%; 94.0%–100.0% | All cases could be measured under the prespecified protocol, including permitted substitution within ±1 mm |
| Candidate levels requiring ±1-mm substitution | 4/180 (2.2%) | 8/300 (2.7%; 7 patients) | 0.6%–5.6%; 1.2%–5.2% | Substitute levels remained within ±1 mm of the target position |
| Adjacent-level correlation coefficient (*r*) | — | 0.86–0.93 | 0.86–0.93 | Indicates substantial information redundancy between adjacent levels |
| Interobserver ICC | 0.949 | 0.954 | 0.916–0.969; 0.925–0.972 | Corresponding to the three- and five-level protocols, respectively |
| Between-protocol agreement | ICC = 0.986 | Three-level vs five-level | 0.977–0.991 | Agreement between the summary results of the three- and five-level protocols |
| STEI, mean ± SD | 2.59 ± 0.50 | 2.60 ± 0.49 | — | Paired measurements in the same 60 patients |
| Paired difference (three-level minus five-level) | −0.009 ± 0.082 | — | −0.030 to 0.012 | Difference close to 0; 95% limits of agreement, −0.170 to 0.152 |
| Per-patient measurement time, min | 6.9 ± 1.2 | 11.0 ± 1.6 | — | Timed by Observer 1 from identification of the reference plane to output of the final STEI |
| Paired difference in measurement time, min | −4.1 ± 0.9 | — | −4.33 to −3.87 | Negative values indicate a shorter measurement time with the three-level protocol |
| Prespecified protocol-finalization criteria | Completion rate ≥95%; interobserver ICC ≥0.90 | Between-protocol ICC ≥0.95; \|paired mean difference\| <0.05; adjacent-level *r* ≥0.85 indicating information redundancy | Measurement time at least 25% shorter with the three-level protocol | All prespecified criteria were met |
| SOP lock point | After completion of the 60-patient technical development set and fulfillment of all finalization criteria | Name, formula, levels, ±1-mm substitution rule, handling of small fragments, and boundary rules locked | Before main-cohort measurement | All 60 development scans were rechecked under the final SOP after lock |

**Note:** The three-level protocol comprised S5, S15, and S25; the five-level protocol additionally comprised S10 and S20. The technical development set was drawn from the 2018–2019 prestudy imaging archive and stratified to include 20 cases each of Schatzker types I–II, III–IV, and V–VI. Review of deidentified images was covered by the same ethics approval and waiver of written informed consent. All measurements were completed without access to FRI outcomes or postoperative treatment information. CI, confidence interval; FRI, fracture-related infection; ICC, intraclass correlation coefficient; *r*, Pearson correlation coefficient; SD, standard deviation; SOP, standard operating procedure; STEI, soft-tissue envelope index.

**Supplementary Table 2.** Proportion and handling of missing data

| **Variable/data item** | **Missing, *n*/denominator** | **Missing proportion** | **Handling** |
| --- | --- | --- | --- |
| STEI data or CT images suitable for STEI measurement | 3/359 | 0.84% | Excluded because the STEI was missing |
| Verifiable outcome assessment after the 12-month postoperative anniversary | 4/359 | 1.11% | Excluded for incomplete follow-up; not assigned to the non-FRI group |
| Albumin | 2/359 | 0.56% | Excluded because one of the four prespecified non-STEI predictors was missing |
| Injury-to-surgery interval | 1/359 | 0.28% | Excluded because one of the four prespecified non-STEI predictors was missing |
| Diabetes status | 1/359 | 0.28% | Excluded because one of the four prespecified non-STEI predictors was missing |
| Three-column involvement data | 1/359 | 0.28% | Excluded because one of the four prespecified non-STEI predictors was missing |
| Other analyzed variables | 0/359 | 0% | No exclusion for missing data |
| Total | 12/359 | 3.34% | 347 patients included in the final analysis |

**Note:** The denominator was the 359 patients who met all other eligibility criteria. Reasons for exclusion were assigned to mutually exclusive categories according to the prespecified hierarchy of missing STEI, follow-up completeness, and the four prespecified non-STEI predictors; each patient was counted under only one primary reason. CT, computed tomography; FRI, fracture-related infection; *n*, number of patients; STEI, soft-tissue envelope index.

**Supplementary Table 3.** Outcome verification beyond 12 months after surgery and final verification source

| **Outcome-verification item** | **FRI group (*n* = 41)** | **Non-FRI group (*n* = 306)** | **Overall (*n* = 347)** | **Description** |
| --- | --- | --- | --- | --- |
| Archived, verifiable outcome record after the 12-month postoperative anniversary | 41 (100.00) | 306 (100.00) | 347 (100.00) | Criterion met by every included patient |
| Actual follow-up duration, months, median (Q1, Q3) | 13.1 (12.4, 14.2) | 12.8 (12.2, 13.8) | 12.8 (12.2, 13.9) | Overall range, 12.1–18.7 months |
| Archived outpatient record from our hospital | 37 (90.24) | 223 (72.88) | 260 (74.93) | Mutually exclusive classification by final primary verification source |
| Routine structured telephone outcome-verification record with complete review of the hospital information systems | 1 (2.44) | 68 (22.22) | 69 (19.88) | Dated standardized checklist generated and archived during clinical care |
| Traceable archived clinical records from an outside institution | 3 (7.32) | 15 (4.90) | 18 (5.19) | Includes outside-institution outpatient, inpatient, and operative records obtained and archived during clinical care |
| Archived outcome confirmation available for non-FRI patients | Not applicable | 306 (100.00) | 306 (88.18) | First-postoperative-year outcome explicitly verified by an archived record after 12 months |
| Non-FRI classification based solely on absence of FRI documentation | Not applicable | 0 (0.00) | 0 (0.00) | An affirmative ascertainment rule was used |

**Note:** Data are *n* (%) unless otherwise indicated. Each patient was assigned to one final verification source on the basis of the archived material closest to the last follow-up time point and with the most complete information after the 12-month postoperative anniversary. The three sources were mutually exclusive and totaled 347 patients. Structured telephone records were generated through the hospital's routine follow-up process using a dated standardized checklist and concurrent review of the hospital information systems. Outside-institution records were traceable documents obtained and archived during clinical care. This study involved only retrospective extraction and review. Standalone imaging or laboratory results served as corroborative evidence. An additional 4 of 359 patients were excluded because no verifiable record was available beyond 12 months. FRI, fracture-related infection; Q1, first quartile; Q3, third quartile; *n*, number of patients.

**Supplementary Table 4.** Distribution of FRI confirmatory criteria

| **Confirmatory criterion** | ***n*** | **Description** |
| --- | --- | --- |
| Sinus tract, fistula, or persistent wound breakdown | 8 | Could overlap with other confirmatory criteria |
| Definite purulent drainage identified at reoperation | 17 | Could overlap with culture or histopathological evidence |
| Concordant microorganism isolated from ≥2 deep-tissue/implant-related specimens | 31 | The same phenotypically indistinguishable microorganism |
| Histopathological evidence of acute inflammation | 11 | Restricted to cases with histopathological specimens |
| Primary-outcome classification based solely on a single positive deep-specimen culture | 0 | A single positive deep culture was treated only as suggestive evidence |

**Note:** FRI, fracture-related infection; *n*, number of patients. Confirmatory criteria could overlap. A single positive deep-specimen culture was not considered confirmatory evidence for the primary outcome.

**Supplementary Table 5.** Reliability analysis of STEI measurement

| **Metric** | **Value** | **Statistical method** | ***P* value** |
| --- | --- | --- | --- |
| Reliability subset | 80 patients: 9 with FRI and 71 without FRI | Stratified random sampling | Not applicable |
| Interobserver ICC | 0.946 (95% CI, 0.920–0.963) | Two-way random-effects, absolute-agreement, single-measure ICC | <0.001 |
| Observer 1 intraobserver ICC | 0.972 (95% CI, 0.958–0.982) | Two-way mixed-effects, absolute-agreement, single-measure ICC | <0.001 |
| Observer 2 intraobserver ICC | 0.958 (95% CI, 0.936–0.973) | Two-way mixed-effects, absolute-agreement, single-measure ICC | <0.001 |
| Interobserver difference | 0.01 ± 0.169 | Bland–Altman analysis | Not applicable |
| Interobserver 95% limits of agreement | −0.32 to 0.34 | Mean difference ± 1.96 × SD | Not applicable |

**Note:** CI, confidence interval; FRI, fracture-related infection; ICC, intraclass correlation coefficient; SD, standard deviation; STEI, soft-tissue envelope index; *P*, two-sided *P* value.

**Supplementary Table 6.** Correlation, sensitivity analyses, and functional forms of continuous predictors

| **Analysis** | **Estimate** | **Statistical method** | ***P* value** |
| --- | --- | --- | --- |
| Correlation between STEI and BMI | Pearson *r* = 0.212 | Pearson correlation analysis | <0.001 |
| Correlation between STEI and injury-to-CT interval | Spearman *ρ* = 0.184 | Spearman correlation analysis | 0.001 |
| STEI effect after adjustment for injury-to-CT interval | OR 1.20 per 0.10-unit increase; 95% CI, 1.11–1.30 | Multivariable logistic regression sensitivity analysis | <0.001 |
| STEI effect after adjustment for BMI and sex | OR 1.20 per 0.10-unit increase; 95% CI, 1.11–1.30 | Multivariable logistic regression sensitivity analysis | <0.001 |
| BMI effect | OR 1.04 per 1 kg/m²; 95% CI, 0.94–1.14 | Multivariable logistic regression sensitivity analysis | 0.440 |
| Male-sex effect | OR 1.18; 95% CI, 0.52–2.70 | Multivariable logistic regression sensitivity analysis | 0.694 |
| Test for nonlinearity of the STEI | *P* for nonlinearity = 0.418 | Restricted cubic spline (3 knots) | 0.418 |
| Test for nonlinearity of injury-to-surgery interval | *P* for nonlinearity = 0.337 | Restricted cubic spline (3 knots) | 0.337 |
| Test for nonlinearity of albumin | *P* for nonlinearity = 0.562 | Restricted cubic spline (3 knots) | 0.562 |

**Note:** Restricted cubic-spline knots were placed at the 10th, 50th, and 90th percentiles. BMI, body mass index; CI, confidence interval; CT, computed tomography; OR, odds ratio; *r*, Pearson correlation coefficient; *ρ*, Spearman rank-correlation coefficient; STEI, soft-tissue envelope index; *P* values are two-sided.

**Supplementary Table 7.** Sensitivity analysis of the three- and five-level STEI measurement protocols

| **Metric** | **Three-level STEI** | **Five-level STEI** | **Statistical method** | ***P* value** |
| --- | --- | --- | --- | --- |
| Mean ± SD | 2.58 ± 0.49 (*n* = 120) | 2.59 ± 0.48 (*n* = 120) | Descriptive statistics | Not applicable |
| Agreement | ICC = 0.982 (95% CI, 0.970–0.989) | — | Two-way mixed-effects, absolute-agreement ICC | <0.001 |
| Paired difference (three-level minus five-level) | −0.0072 ± 0.0940 | — | Paired *t* test, *t* = −0.84 | 0.403 |
| AUC | 0.791 | 0.796 | DeLong test | 0.684 |

**Note:** The three-level protocol comprised S5, S15, and S25; the five-level protocol additionally comprised S10 and S20. The paired difference, SD, *t* statistic, and *P* value are all based on *n* = 120. AUC, area under the receiver operating characteristic curve; CI, confidence interval; ICC, intraclass correlation coefficient; *n*, number of patients; SD, standard deviation; STEI, soft-tissue envelope index; *P*, two-sided *P* value.
